# Importance of biomarkers in glioblastomas patients receiving local BCNU wafer chemotherapy

**DOI:** 10.1186/s13039-017-0317-5

**Published:** 2017-05-04

**Authors:** Steffi Urbschat, Christoph Sippl, Jana Engelhardt, Kai Kammers, Joachim Oertel, Ralf Ketter

**Affiliations:** 1 Department of Neurosurgery, Saarland University, 66421 Homburg/Saar, Germany; 2 Division of Biostatistics and Bioinformatics, Department of Oncology, Sidney Kimmel Comprehensive Cancer Center, Johns Hopkins University School of Medicine, Baltimore, MD USA

**Keywords:** Glioblastoma, CGH, Carmustin wafer, Prognostic factors, Standard combined radiochemotherapy

## Abstract

**Background:**

To assess the influence of molecular markers with potential prognostic value to groups of patients with newly diagnosed glioblastoma patients were examined: group A with 36 patients (surgical resection plus standard combined chemoradiotherapy) and group B with 36 patients (surgical resection, standard combined chemoradiotherapy plus carmustine wafer implantation).

Our aim was to determine chromosomal alterations, methylation status of MGMT, p15, and p16 (CDKN2A) in order to analyse the influence on patient survival time as well as radio- and chemotherapy responses. Promoter hypermethylation of MGMT, p16, and p15 genes were determined by MS-PCR. Comparative genomic hybridisation (CGH) analyses were performed with isolated, labelled DNA of each tumor to detect genetic alterations.

**Results:**

Age of onset of the disease showed a significant effect on overall survival (OS) (*p* < 0.0001). Additional treatment with carmustine wafer (group B) compared to the control group (group A) did not result in improved OS (*p* = 0.562). Patients with a methylated MGMT promotor showed a significant longer OS compared to those patients with unmethylated MGMT promotor (*p* = 0.041). Subgroup analyses revealed that patients with methylated p15 showed a significant shorter OS when administered to group B rather than in group A (*p* = 0.0332). In patients additionally treated with carmustine wafer an amplification of 4q12 showed a significant impact on a reduced OS (*p* = 0.00835). In group B, a loss of 13q was significantly associated with a longer OS (*p* = 0.0364). If a loss of chromosome 10 occurred, patients in group B showed a significantly longer OS (*p* = 0.0123).

**Conclusion:**

A clinical benefit for the widespread use of additional carmustine wafer implantation could not be found. However, carmustine wafer implantation shows a significantly improved overall survival if parts of chromosome 10 or chromosome 13 are deleted. In cases of 4q12 amplification and in cases of a methylated p15 promotor, the use of carmustine wafers is especially not recommended.

The MGMT promoter methylation is a strong prognostic Biomarker for benefit from temozolomide and BCNU chemotherapy.

## Background

Glioblastoma multiforme (GBM) is the most aggressive and most common form of primary brain cancer [1]. At an incidence of 3 to 4 in 100,000 and a slight male predominance of 1.2–1.9:1 it can strike all ages [2–4]. The gold standard treatment for GBM is a grand total resection combined with radiochemotherapy consisting of 60 Gy radiation and temozolomide (TMZ) according to Stupp et al. [5]. Another option in the treatment of GBM is, in addition to the Stupp-regime, the implantation of carmustin (BCNU) eluted wafer into the resection cave. Hence a higher concentration of anti-neoplastic agents can be released to the tissue adjacent to the tumor. This strategy minimizes the systemic effects and maximizes the anti-neoplastic effect by bypassing the blood brain barrier [6]. Despite this theoretical advantage, the clinical benefit remains in dispute. Some authors showed a benefit regarding overall survival (OS) when carmustin wafers were implanted postresectionally [7, 8]. In contrast Pallud et al. could not show a long-term benefit in (OS) at a cohort of 354 patients [9]. However, some adverse effects like cerebral edema and postoperative wound infection can be increased [10]. Therefore, it would be eligible to predict the response characteristics of a carmustin wafer therapy. Whether carmustine wafer implantation is recommendable or not could depend on patients’ individual (genetic) characteristics.

A marker, which is known to predict chemotherapy response in GBM, is the promotor methylation status of O6-methyl-guanine-methyl transferase (MGMT), a DNA repair enzyme. This protein repairs alkylating DNA damage induced by TMZ in tumor cells and hereby promotes tumor progression of GBM [11]. Several clinical studies showed that low MGMT expression was significantly related to ameliorated TMZ therapy response [12–19]. Carmustine as the active drug in carmustine wafer is also a DNA alkylating agent, which can be counteracted by MGMT [20]. Another potential marker for the clinical course of GBM is a hypermethylation status of p15 that was shown to be associated with a shorter OS [21].

Furthermore, chromosomal alterations like gains on chromosome 7 and losses of parts of chromosome 10 and of/parts of the short arm of chromosome 9 are common phenomenon in glial tumors. Especially losses on chromosome 9p and 10q are often associated with a poor prognosis for patients with GBM [22]. Losses of chromosome 9 affects p16, a cell cycle controlling protein located on 9p21.3.

The aim of this experimental trial was to find new markers for treatment response in GBM on genetic and also epigenetic levels and to investigate the effects of additional carmustine wafer therapy on known markers. In particular, we focused on genes regulating cell cycle, e.g., p15, p16, and the DNA repair enzyme MGMT. For further stratification and subgroup analyses, we also incorporated two different treatment modalities: one patient cohort treated according to Stupp et al. and the other patient cohort received carmustine wafer implantation after resection in addition the Stupp et al. regime [5].

## Methods

### Patients

In this trial, we enclosed 72 patients with newly diagnosed GBM who underwent surgery between 2005 and 2012 at the department of neurosurgery at the university medical center Homburg/Saar, divided into two matched pair groups with 36 patients each. After tumor resection one group was treated according to the standard Stupp regime (group A), whereas the other cohort was treated according to the standard Stupp regime with carmustine wafer implantation (group B).

Tumor tissue was collected at the time of surgery, if clinically indicated. All tissue samples were frozen immediately after the tumor was resected using liquid nitrogen and stored in our tumor bank at −80 °C. This study was approved by the local ethic board of Saarland and written informed consent was obtained from all patients.

### Methylation analysis

DNA isolation was performed using DNA isolation kit (Qiagen, QIAamp DNA Mini Kit 50). The methylation status of promoter regions of the genes p15, p16 and MGMT were determined by methylation specific polymerase chain reaction (MS-PCR). Therefore, 500 ng DNA of each tumor specimen, as well as appropriate control samples were bisulfite-treated (ZYMO RESEARCH, EZ DNA Methylation-Gold Kit 200) [23]. In summary, thus unmethylated cytosine is converted to uracil, whereas methylated cytosine remained unchanged. The modified DNA was recovered by ethanol precipitation and dissolved in water. For the analysis of the methylation status the primer sequences listed in Table 1 were used.

**Table 1:** Primer for MS-PCR

p15 [22]: (methylated, 148 bp, 60 °C) forward: 5′-GCGTTCGTATTTTGCGGTT-3′ reverse: 5′-CGTACAATAACCGAACGACCGA-3′ (unmethylated, 154 bp, 60 °C) forward: 5′-TGTGATGTGTTTGTATTTTGTGGTT-3′ reverse: 5′-CCATACAATAACCAAACAACCAA-3′	p16 [23]: (methylated 150 bp, 65 °C) forward: 5′-TTATTAGAGGGTGGGGCGGATCGC-3′ reverse: 5′-GACCCCGAACCGCGACCGTAA-3′ (unmethylated 151 bp, 65 °C) forward: 5′-TTATTAGAGGGTGGGGTGGATTGT-3′ reverse: 5′-CAACCCCAAACCACAACCATAA-3′
MGMT [21]: (methylated, 122 bp, 54 °C) forward: 5′-GTTTTTAGAACGTTTTGCGTTTCGAC-3′ reverse: 5′-CACCGTCCCGAAAAAAAACTCCG-3′ (unmethylated, 129 bp, 56 °C) forward: 5′-TGTGTTTTTAGAATGTTTTGTGTTTTGAT-3′ reverse: 5′-CTACCACCATCCCAAAAAAAAACTCCA-3′	

**Fig. 1:**
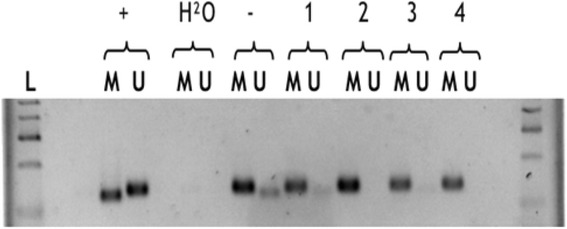
Methylation specific PCR of MGMT promotor in GBMs. L = Molecular size marker; U = unmethylated DNA; M = methylated DNA; + = positive control; − = negative control; H_2_O = blank value; 1 = case 1442/08; 2 = case 1510/10; 3 = case 1219/19; 4 = case 288/08

PCR was performed with a 25 μl reaction volume and 38 PCR cycles. All PCR products were separated by electrophoresis on a 2% agarose gel. As methylated and unmethylated control we used Universal Methylated Human DNA (ZYMO RESEARCH). As blank value we added water in place of DNA [Fig. 1].

### CGH analysis

Comparative genomic hybridisation (CGH) was used to screen the tumors for chromosomal imbalances. Metaphasic preparation was acquired via short term lymphocytic culture. CGH was performed as described previously [24].

### Statistics

Comparisons of survival times between groups defined by clinical variables, methylation status and parts of chromosome deletions were performed by Kaplan-Meier curves and with two-sided log rank tests. Methylation index was defined as percentage of patients with promotor methylation of the total cohort in percent. Univariate Cox regression [25] analysis was performed to identify significant predictors for overall survival (OS). Effects of individual predictors on OS in all models were quantified by estimated hazard ratios (HR) estimates with corresponding 95% confidence intervals.

## Results

### Clinical data

Overall median survival was 267 days (95% CI = [176, 372]) with 309 days in group A (95% CI = [138, 481]) and 219 days in group B (95% CI = [151, 372]).

Univariate Cox models of clinical covariates revealed that age of onset of the disease had a statistically significant effect on OS (HR = 1.048, 95% CI = [1.024, 1.072], *p* < 0.0001). The average age of onset was 60.1 years in group A and 60.2 years in group B. Gender did not show a significant effect on OS (HR =1.19, 95% CI = [0.715–1.977], *p* = 0.504) (see Table 2).

**Table 2:** Clinical, epigenetic and CGH results (univariates Coxmodel)

	HR	1/HR	lover.95	upper.95	*p* value
Clinical data					
groupe	1.1561	0.8649	0.7079	1.8880	*p* = 0.5620
age	1.0479	0.9542	1.0242	1.0721	*p* = 0.6081
gender	1.1889	0.8410	0.7151	1.9767	*p* = 0.5046
Methylation					
MGMT	0.5929	1.6866	0.3590	0.9789	*p* = 0.041
p15	0.7883	1.2684	0.4065	1.5287	*p* = 0.4816
p16	1.5724	0.6359	0.6735	3.6711	*p* = 0.2954
Comparative genetic hybridization [CGH)					
1q	2.1684	0.4611	0.6683	7.0355	*p* = 0.1974
amp4q12	2.3050	0.4338	1.1815	4.4965	*p* = 0.01431
7	1.5579	0.6418	0.7591	3.1973	*p* = 0.2267
amp7p12	1.4766	0.6771	0.8132	2.6813	*p* = 0.2002
9p	1.0812	0.9248	0.6633	1.7626	*p* = 0.7538
10	1.0692	0.9352	0.6536	1.7490	*p* = 0.7897
10q	0.8369	1.1948	0.4945	1.4162	*p* = 0.5071
12q	1.0097	0.9903	0.5659	1.8015	*p* = 0.9737
13	0.6853	1.4591	0.4169	1.1264	*p* = 0.1361
17	1.0081	0.9919	0.5704	1.7816	*p* = 0.9778
20	1.4714	0.6796	0.8380	2.5835	*p* = 0.1787
22	1.0570	0.9459	0.5908	1.8912	*p* = 0.8515

**Fig. 2:**
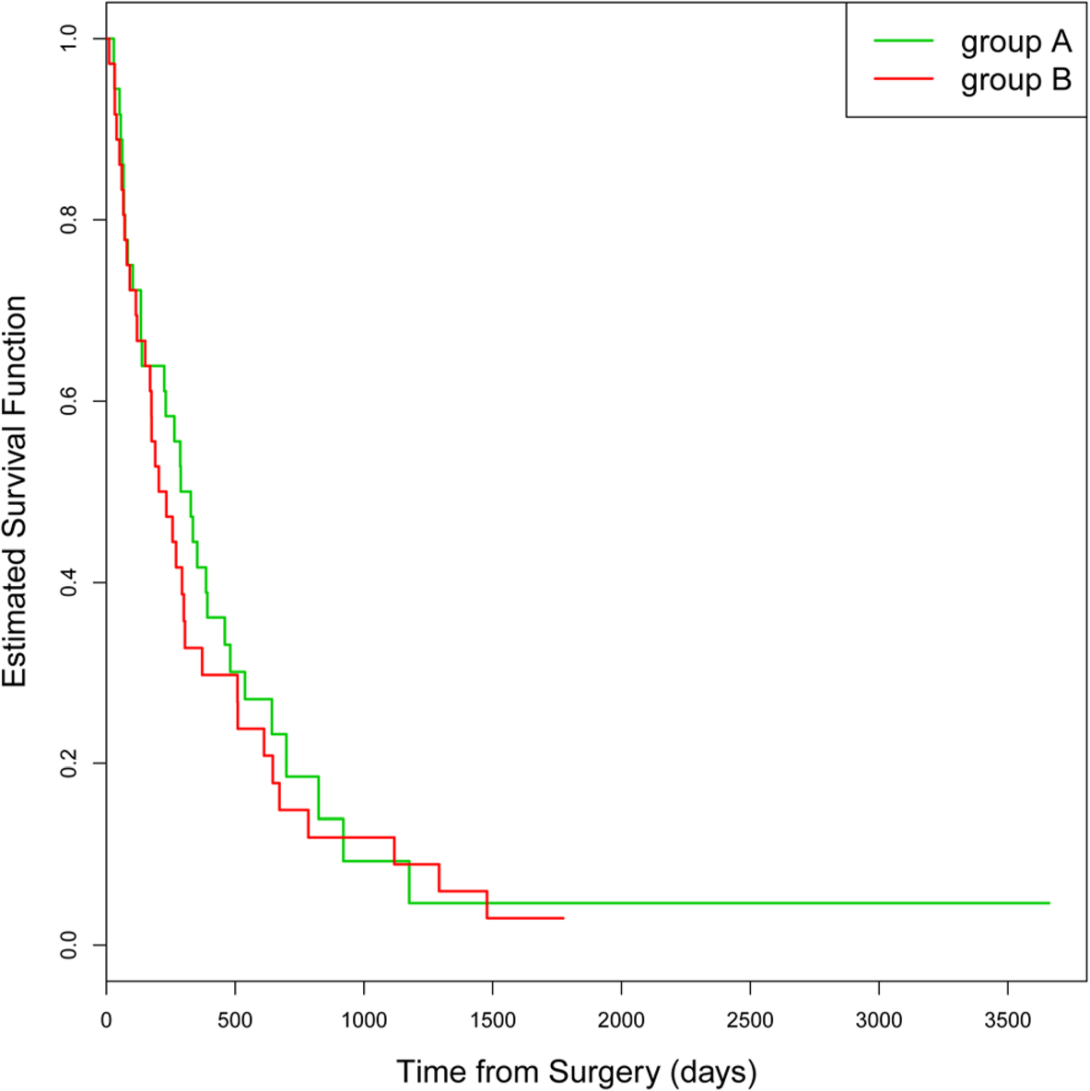
OS in group A (standard Stupp regime) and group B (standard Stupp regime + carmustine wafer)

A statistically significant effect of additional treatment with carmustine wafer (group B) in comparison to the standard Stupp regime (group A) on OS could not be detected (HR = 1.15, 95% CI = [0.708, 1.888], *p* = 0.562) [Fig. 2].

### Methylation analysis

We found a MGMT methylation index (MI) of 58% (21/36) in group A and a methylation index of 42% (15/36) in group B. At the p15 promotor we found a MI of 14% (5/36) in group A and 25% (9/36) in group B, respectively. The methylation index in p16 showed 8% (3/36) in both groups.

**Fig. 3:**
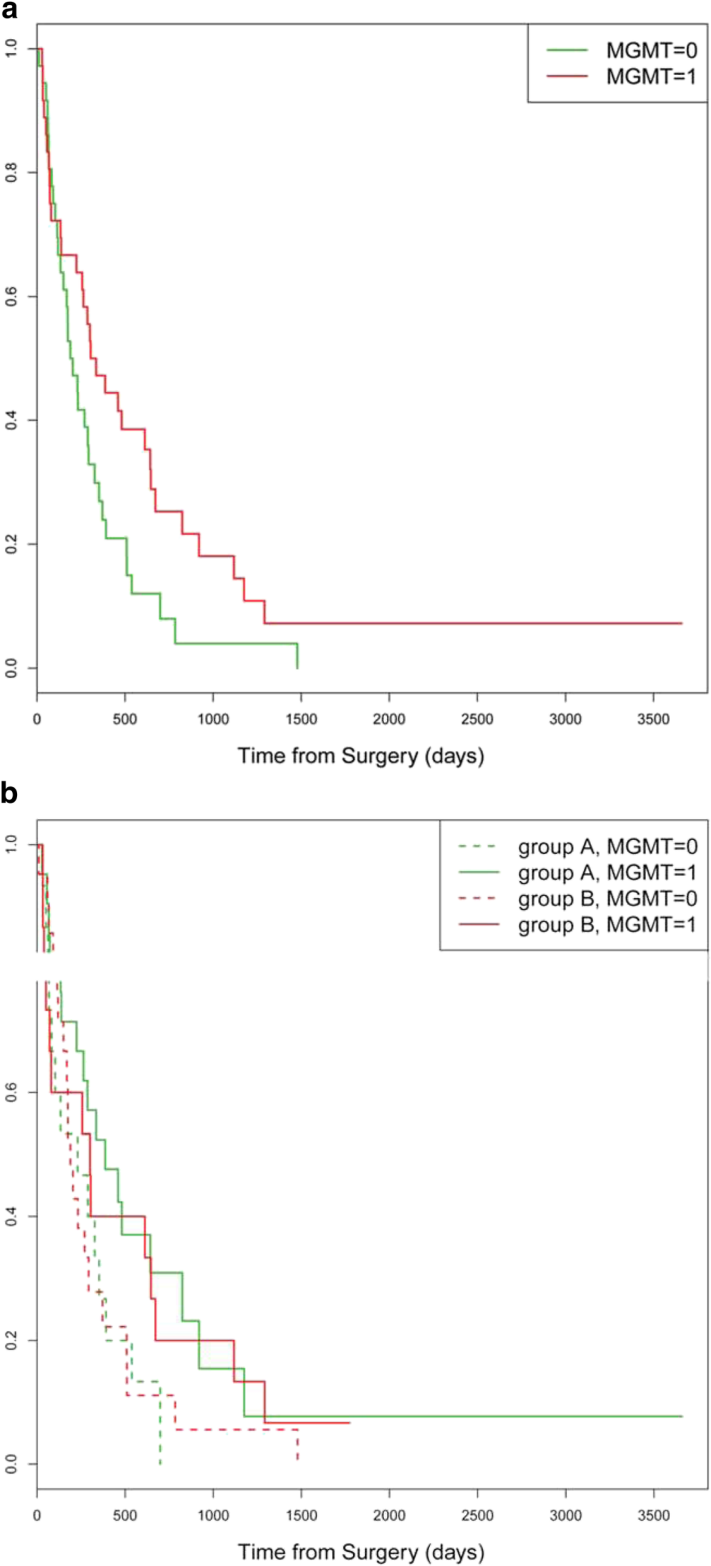
**a** OS depending on MGMT methylation status. *Green*: no methylation of MGMT promoter. *Red*: methylation of MGMT promoter. **b**: OS in group A and B depending on MGMT methylation status. 0: no methylation of MGMT promoter. 1: methylation of MGMT promoter

Patients with an unmethylated MGMT showed a median OS of 6.6 months. If MGMT was methylated the median OS was 10.7 months. A univariate Cox model with MGMT as predictor results in MGMT has a statistically significant effect on OS (HR = 0.593, 95% CI = 0.359 – 0.979, *p* = 0.041). If stratified for treatment group there was neither in group A nor in group B a significant correlation between OS and MGMT methylation (group A: *p* = 0.0635, group B: *p* = 0.319) [Fig. 3a/b].

**Fig. 4:**
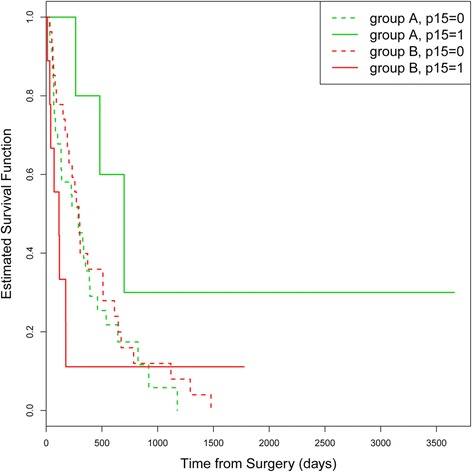
OS in group A and B depending on p15 methylation status. *Green*: group A (Stupp regime). *Red*: group B (Stupp regime + carmustine wafer)

Subgroup analyses revealed that patients with a p15 methylation showed a significant shorter OS when administered to group B (median OS: 115 days) than in group A (median OS: 481 days) (*p* = 0.0332). A promotor methylation of p16 had no significant impact on any group [Fig. 4].

Within the whole population in this study (*N* = 72) 3 patients showed an OS longer than 36 months, 2 of them were in group B, one in group A. All three cases showed a methylated MGMT promotor whereas p15 and p16 were not methylated.

### CGH

**Fig. 5:**
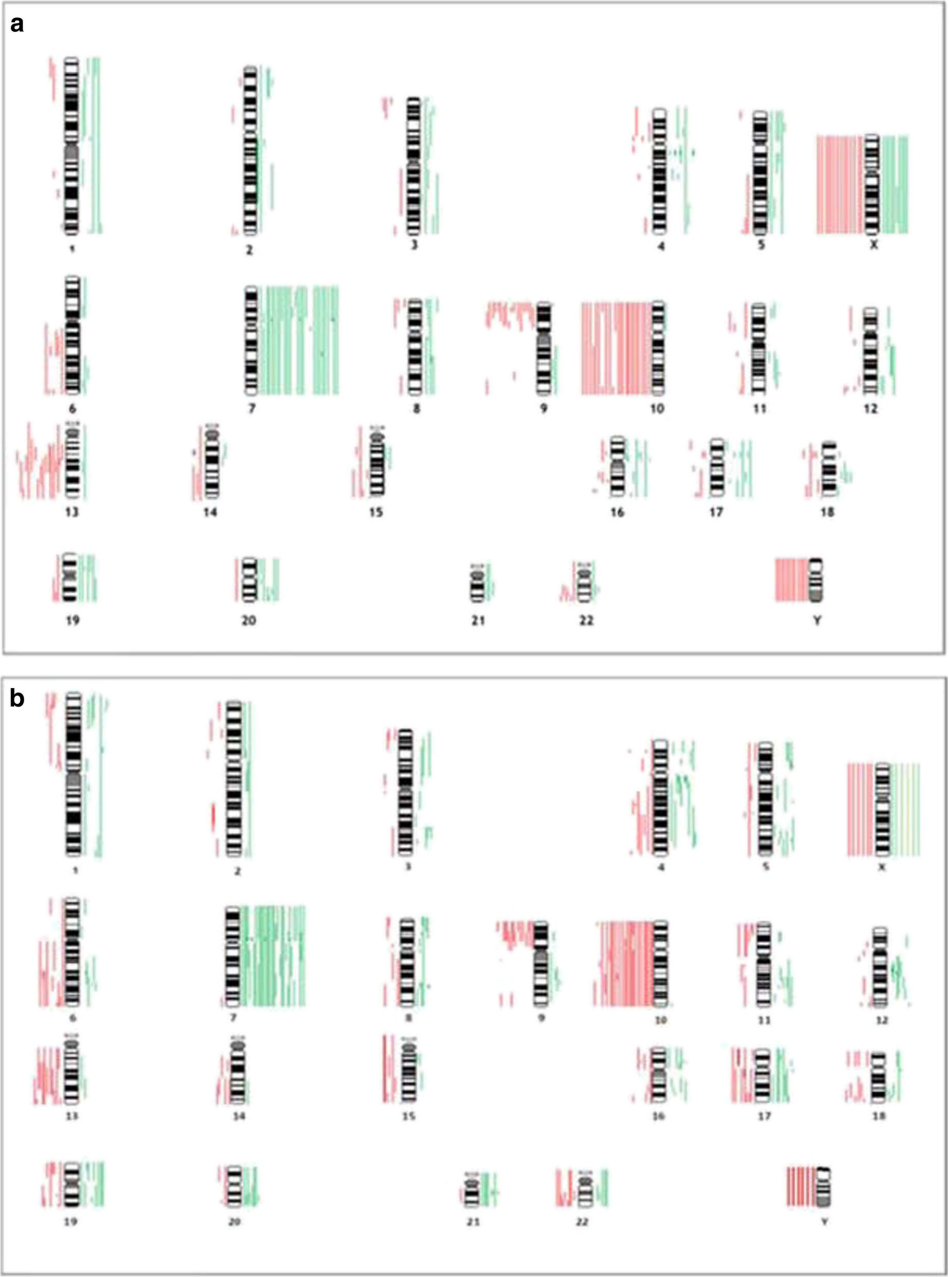
**a** Overview of genetic imbalances of the Carmustin-group. Lines on the left represent losses, and lines on the right represent gains; amplifications are in bold. **b**: Overview of genetic imbalances of the conventionally treated-group. Lines on the left represent losses, and lines on the right represent gains; amplifications are in bold

In total, each tumor showed on average 11 aberrations and a total number of 754 aberrations could be detected. The distribution of alterations in both groups showed in general no differences [Fig. 5 a/b]. We found different chromosomal alterations in all the analyzed tumor specimens. One of the most frequent alterations were gains on chromosome 7 in 85% (61/72), chromosome 16 in 33% (24/72), chromosome 4 in 22% (16/72), chromosome 5 in 21% (15/72), chromosome 12 in 19% (14/72) and chromosome 20 in 22% (16/72).

In contrast losses were prevalently detected on the short arm of chromosome 9 in 47% (34/72), chromosome 10 in 67% (48/72), the long arm of chromosome 13 in 47% (34/72), on chromosome 6 in 25% (18/72), on the long arm of chromosome 22 in 22% (16/72), of the long arm of chromosome 4 in 19% (14/72) and on the long arm of chromosome 17 in 18% (13/72) [Fig. 5 a/b].

We also found amplifications at 4q12 in 15% (11/72), at 7p12 in 19% (14/72) and in the region of 12q in 4% (3/72). In one case an amplification of 6q21 and in another case of 8p23.1pter were found (Tables 3, 4 and 5).

**Table 3:** Clinical chacteristics and CGH results of the Carmustin group

Cases	Histology	Ages/gender	CGH - results
2065/08	pGBM	80/f	+X, +7, −9p, −10, −14q24qter, −17p12pter, −22q11.2q13.2
270/10	pGBM	80/m	−4, −6, +7, −8q21.1qter, +9q12q34.1, −10, −11p14pter, −16, −17q, −18p11.2pter, +19, +20, +21
1526/07	pGBM	75/m	-X, +1p33pter, −1p11p22.1, amp4q12, +6p12p21.3, +7, −9p13pter, −10, +11q23.3q25, +15q11.2q14, +16p, +17p12pter, +20q12q13.2
220/10	pGBM	72/f	+X, −6q25.3qter, +7p, amp7p12, +7q11.1q31.1, −9p23pter, −10, −11p15.3pter, −13q33qter, +17, −18q22qter, +21, −22
479/09	pGBM	69/f	+5q23.3q32, +7q21.1q36
740/10	pGBM	69/f	−2p16p21, −3p24.1pter, −3p12p13, −3q24qter, −4q13.3q33, +7q11.2, −9p21p23, −11p14p15.3, −15q21.3q25, −16q13q22, +17, −18q12.3q22
1442/08	pGBM	52/f	+4q13.1q32, +7q, -10, +12q13.3.q15, −19, −22
1578/10	pGBM	52/f	+1q, +4p15.3pter, +5q12q14, −6q23.1qter, +6p22.3pter, +7, −8p11.1p12, −8q12q21.3, −9p21pter, −10, +11p14p15.3, −12q24.1qter, +15q11.1q15, +17p12p13, −17q25qter, +19p13.3pter, +19q, +20, +21, +22q11.1q13.1
1966/11	pGBM	40/f	-X, +3q21q24, amp7p12, +7q22qter, −17q25qter, +22q12.1q13.1
2404/11	pGBM	75/m	−2p23pter, −4p15.3p16, +7, +9p21p23, −10p13pter, −10q, +12q13.1q21.2, amp12q14, −12q24.3qter, −16p11.2p13.2, −19q13.3qter, −22q12.1q13.1
1210/09	pGBM	71/m	−4q28q32, −8p21.1pter, −9p21pter, −11p, −13, −15q12q21.3, −15q26.1qter, +16q11.2q21, −18p, −18q12.1qter, +19p, −20
1510/10	pGBM	69/m	+1p32.3p36.1, −3p24.1pter, +3q26.1q26.3, +4q24q34, −5p14pter, +5q31.1q32, +6q16.1q22.3, amp6q21, −6q27, +7, −9p22pter, −10, −11p, −11q14.1qter, +12q12q23, amp12q14q15, −13q23qter, −15q25qter, −16p12p13.1, +16q21q22, −18p, −18q23qter, +19q13.3, +20q11.2q12.3, +21, +22
1370/07	pGBM	63/m	+1p11p22.1, +2p11.1p33, +7, amp 7p12, −3p21.3p25, −9p13p23, −9q31qter, −10, −17, −19p13.2pter, −20q13.1q13.2, −21q22.1q22.2, −22q13.1qter
1663/07	pGBM	58/m	-X, +1p34.1pter, −1p21p31.1, −2q24.1q32.3, −4p11p15.1, −4q11q28, −5q21q22, +5q32qter, −6q11q21, +7, +8q24.1qter, −9p21pter, −10, −12p11.2p12.3, −12q11q12, +12q15q22, −13q14.3q31, −14q12q21, +17, +20q, +22
1219/09	pGBM	58/m	−1p13.1p13.2, −6q22.2q24, +7, amp7p12, +11q14.3q22.3, −12q24.3qter, +18q21.3q22, −19p13.2pter, +21p11.2p21
1809/11	pGBM	55/m	−1p26.1pter, +3p12p22, amp4q12, +5p14pter, +5q11.2q21, -6p22.3pter, +6q22.2qter, +7, +8p11.2p21.1, +8q11.2, +8q22.3q23, −9p, +9q21.3q22.3, −9q34.1qter, −10p11.1p12.3, −10q, +11p14p15.3, +11q13.2q14.3, +12q12q23, −13q11q32, -14q22qter, −15
1362/07	pGBM	52/m	−1p34.3pter, −5q12q14, +7, −9p11p22, −10, +18q21.3qter, +19p
288/08	pGBM	52/m	amp7p12, +7q31.3q35, +9q21.2q32, −10, −11q12q13.2, -13q12.3q31, −17q11.1q21.1
1350/06	sGBM	46/m	+1q12q23, amp4q12, +4q12q22, +8q23qter, −1p21p36.1, -4q28qter, −6q, −13q14.3q33, −18q12.3q23, −19q13.1q13.3, -22q12.3qter
1455/08	pGBM	46/m	+4p14p15.1, amp4q12, +7p, +8p21.2p22, +11q14.1q22.3
768/09	pGBM	44/m	−6q25.1qter, +7q21.2q32, +12q13.2q21.1, −18p, −19
P.E.	pGBM	44/m	+1q43qter, +2p11.1p13, +2q13q22, +2q37.1qter, +3p13p33, +4p15.3pter, +5p14pter, +5q31.1qter, +6q23.1q22.3, +7, +9q22.3q31, −10q22.1q22.3, +11q23.3, +12q15q21.3, −13, +16q23q24, +17q12q24, +18q22
1741/08	pGBM	54/f	−9p21pter, −10, −17q, +18q12.3q22
1701/07	pGBM	67/m	amp 4q12, +7, +17q, −4q21.3q34, −5, −10, −11q12q21, -13q14.1qter, −17p, −19p11pter
1935/08	pGBM	70/m	amp4q12, +7q11q21.3, −9p21pter, −10, −18q22qter, −20p11.2pter
1618/09	pGBM	54/f	−2q24.1q34, −3p11.1p14.1, −3q11.1q13.1, −4q24q26, −5p11p12, -6p11p12, amp7p12, +7q34qter, −10q11.1q21.1, +10q26.2qter, -13q14.1q31, −14q12q22, +16p, +17q24qter, +19q, +22
A.W.	pGBM	75/m	+3q24q27, +4p, +5p, amp5p﻿13.1p14, +5q32, +7, amp7p12, −9p21pter, −10, −12q12q13.1, −13q21.3q33, +14q23qter, +15q21.1q24, +17p11.2p12, +19, +20, +21q11.2q21
1934/11	pGBM	69/f	+X, +3q24q26.1, +4q13.1q32, +6q15q22.3, +7q21.1q32, −9q13q21.1, +11q14.1q22.3, +12q21.3q24.1, −16p, −17p, −19q13.2qter, −22
1381/06	pGBM	44/m	-X, −Y, +1q32.2qter, −2p15p23, +2q11.1q21.3, −4q24q28, −6q21q22.3, +7p, +7q33qter, +8, amp 8p23.1pter, −10q22.1q25.1, −11p, +11q, −13q11q31, −14q22q31, −21q11.1q22.1, −22
1080/10	pGBM	73/m	+X, −1p34.1pter, +4p14p16, +6q23.1q24, +7p, +8p22pter, −9q34qter, −10, +12p11.2p13.1, +16p11.2p12,-16q24qter, −17q, +18p11.2pter
725/12	pGBM	50/m	-X, +1, +2, +3p13p25, +3q24, +4p, +4q26q34, +5p13.1p13.3, +5q14q15, +5q23.1q34, −6q, +7, +8p21.1pter, +8q23, -10q21.1q21.3, +11q14.1qter, −13q33qter, −14q24.2qter, +16p, +16q11.2q22, +18, +19, +20, +21
778/10	pGBM	70/m	+1p36.1, −4q34qter, amp7p12, +7q11.1q22, -10p15pter,+12q24.3qter, −13q23.1qter, +17p11.1p11.2, +18p11.1p11.2, +19,+20q12q13.2
563/12	pGBM	60/m	−7q36qter
904/11	pGBM	65/m	-X, +1p36.1p36.3, −4p16, −9p23pter, +16p12p13.1
1346/06	pGBM	50/f	−5q21q23.1, −8q23qter, −10q23.2qter, −15, −17
175/12	pGBM	45/m	-X, +1p31.1p32.3,-2p11.2p13, +3q24q26.1, +4q31.3q34, +7, +8q23q24.1, +16q12.2qter, +16p12p13.2, −19q, +21q11.2q22.1

**Table 4:** Clinical characteristics and CGH results of the Controll group

Cases	Histology	Ages/gender	CGH-results
1553/07	pGBM	82/f	+2q12q33, +3p24.2p25, +3q22q24, amp4q12, +7, −9p13p24, −10, +11q13.2q14.1, −13q12.1q14.3,-15q21.3q23, +16p, +17p12pter, +18q23qter
1042/12	pGBM	83/f	−2p23p24, −4p12p15.1, −4q21.2q22, amp7p12, +7q, −10, −13q14.1q21.1, −16q13q21, −18q12.3q22
1117/05	pGBM	71/m	+3, +7, −9p21pter, −10, −13, −14, +16p11.2p13
139/10	pGBM	73/f	+1p13.1p33, +1q21.1q31, +2, +3p12p14.1, +3p25pter, +3q13.1q21, +4q22q24, +5p14, +7, +8p23.1pter, +8q11.2qter, +9p23pter, +9q22.1q33, −10, +13q14.1q21.3, +14q12q21, −16p12p13.1, +16q23qter, −17p11.1p11.2, −18p11.2p11.3, −19, −22
1588/09	pGBM	70/f	+X, +1p21p36.1, +3p14.1p24.1, −3q11.1q26.1, +5q23.2q32, +7, amp 7p12, −9p22pter, −10, +12q21.3q24.3, −17p11.1p11.2, +17q21.3q22, −18p11.1p11.3, +19, +20
1191/08	pGBM	68/f	+1p36.1pter, −1q42.2q43, amp4q12, +7, −10, -13q14.1q21.3, +16q11.2q21, −17q12qter, +18p11.1p11.2
1355/08	pGBM	52/f	-X, −1q31, +6q24q26, +7, −8p21.1pter, −9p22pter, −10, +11p15.1p15.4, +12q21.3qter, −13q12.2qter, +15q11.1q15, +16, −18, −19q13.2qter, +20p, +20q11.1q12, +21, −22q13.2qter
922/12	pGBM	54/m	+1p13.1p13.3, +1q43qter, +2q12q14.1, -2q37.1qter, +4p13pter, +4q24q25, +5, +7, +8p12pter, +8q12q13, -10, +11p15.1pter, +11q13.1q23.3, -13q12.1q21.3, +16p12p13.1, +17p11.1p12, +19q13.1q13.2, +22
881/10	pGBM	40/f	+1, +2p21pter, −5q14qter, −6q14q21, −9p24pter, −9q31q33, −10, −11p, −12q12q13.1, −12q24.1q24.3, -13q14.1q21.2, +16p11.2p13.1, +17q21.1qter, +22q11.2q12.3
1229/08	pGBM	72/m	−1p31.2p36.1, −1q23q24, +3q26.1qter, amp4q12, +4q11q21.2, −4q33qter, −6q13q21, +7, −9p23p24, −10, −11q14.1qter, −13q14.1qter, −14q22q32.1, +16, +17p11.1p12, −22q13.1q13.2
375/10	pGBM	72/m	+1p36.1pter, +2p21pter, −4p11p12, +6p12p21.3, −9p13p21, −13q21.1q31, +15q13q21.3, +17, +20q12qter
1988/09	pGBM	68/m	amp7p12, −10p, +11q13.5q23.3, −12p, +20q31.1qter, +22q12.3q13.1
838/12	sGBM	70/m	+7, −9p, −10, +19
926/09	pGBM	58/m	−3q27qter, −6q26qter, +7, amp7p12, −8p23.1pter, −9p13pter, −10, −16p, +16q11.2q21, −18q21.3qter, +19, +20q12q13.2, −22q12.3qter
983/04	pGBM	54/m	−3p24.3pter, −4q23q25, −6q16.3q21, −8q24.1qter, −10, −11q23.3qter, −13q22qter, −17q25qter
837/12	pGBM	54/f	+7, −8p22pter, −9p13p24, −10, +17q21.2q22, +18q12.1q12.3, +19p
1078/09	pGBM	54/m	+Xp, +Xq11.1q21.3, +1, −3p21.3pter, +3q13.2qter, −4p14pter, +4q28q34, +5p, +5q11.2q13.2, −5q33.1qter, +6p, +6q21qter, +7, +8, −9p23pter, +12p, +12q11q13.1, −13, −14q21qter, amp 14q11.2q12, +17, +19, −20, +21
776/05	pGBM	50/m	+3p23p24.3, +5q23.3q31.1, +7p14p22
1861/03	pGBM	43/m	+2p23p24, +2q22q34, +6q22.3q24, +7, −8p, −9p23pter, −10, amp12q13.12q21.3, +13, −15q12q14, +18q12.1q21.2, −22q11.2q13.1
1216/11	pGBM	50/m	+7p11.1pter, amp7p12, −9p21pter, −10q21qter, +11q21q22.3, +12q13.1qter, −13q14.1q21.3, −22q13.3qter
1110/05	pGBM	41/m	+7p
1457/03	pGBM	37/m	−5q34qter, +8q21.3qter, +10p11.2pter, −12q24.1q24.3, −13q33qter, +18q12.2q21.1
378/12	pGBM	59/f	+1q42.2qter, −4p11p13, −4q11q13.1, +5p14p15.2, -6q, +7, amp7p12, +8p22pter, −9p13pter, −10, −11p12p14, −13q14.3q33, +14q11.1q13, −16q23q24, −17p13pter, −18p11.1p11.3, +19q, +20, +21q11.1q22.1
1583/08	pGBM	68/f	−2p23p24, −5q21q31.1, +5q33.1qter, −6q12q14, +7, +8p23.1pter, −8p11.1p12, −9q22.3qter, −10, −13q33qter, +16p11.2p13.1, +17p11.1p12, +19p13.1p13.3, +20, +21q11.2q22.1, −22q11.2qter
655/09	pGBM	71/f	−6q12qter, +7, −9p21pter, −10, −12q21.3q23, −13q12.3qter, +20
291/09	pGBM	51/f	+4p14, −6q26qter, +7p13pter, +18q11.1q21.1, +Xq21.2q27
156/12	sGBM	76/f	-1p34.2pter, +5, +7, +9q13qter, −15q21.3qter, −16q11.2q13, −17q11.2q21.1
1286/08	pGBM	65/m	+7, −10q25.1qter, +16p12
1458/10	pGBM	49/f	+7q11.2, −13q12.3q14.1, −15q26.1qter
1431/05	pGBM	69/f	−2p12p15, −2q37.1qter, −3p24.1pter, amp4q12, −5p15.3pter, +7, −9p13p23, −9q33qter, −10, −11p12p15.3, −12q24.1qter, −13q13q21.1, −16q23q24
T 6929	pGBM	50/m	+1p21p31.2, +3q12q13.2, +3q25.3qter, −4p16pter, +6q12q13, +7, −9p21p23, −9q13q23.1, −10, −11q23.1q23.3, −12q22qter, −13q12.3qter, −17, −18p11.1p11.3, −20, +Xq13q23
338/12	pGBM	72/m	-X, +7, −9p21pter, −11q12q13.2, +17p11.2p12, +20
791/08	pGBM	56/m	No aberrrations
663/12	pGBM	65/f	+4, +7, −10p14pter, −10q25.2qter, +12q13.1q13.3, −15
754/12	pGBM	56/m	+5p, +5q11.2q14, +7, −10, −13q11q21.1, −17q24qter, −19q
2512/11	pGBM	42/m	-Xp, +4q, amp4q12, −5q13.3qter, −7p14pter, +7q, +8p, −9q34.1qter, −10q22.3qter, −11p, −11q24qter, −12p, −12q14q23, −13, −18p11.2pter, −18q22qter, −21

**Table 5:** Overview of the chromosomal alterations

Alteration	Group B frequency	Group A frequency
amplification 4q12	6/36 (17%)	5/36 (14%)
gain on chromosome 7	30/36 (83%)	31/36 (86%)
amplification on 7p12	8/36 (22%)	6/36 (17%)
loss on 9p	16/36 (44%)	18/36 (50%)
loss on 10q	23/36 (64%)	25/36 (69%)
gain on 12q	9/36 (25%)	6/36 (17%)
loss of chromosome 13	13/36 (36%)	21/36 (58%)
loss of chromosome 20	8/36 (22%)	9/36 (25%)
loss of chromosome 22	8/36 (22%)	7/36 (19%)

Patients in group B whose tumors showed an amplification of 4q12 had a statistically significant reduced OS (log-rank test, *p* = 0.00835). An amplification of 4q12 for patients of group A did not show this worsening effect on OS. In contrast if a loss of chromosome 10 occurred in tumor samples, patients in group B, who additionally received carmustine wafer implantation, showed a significantly longer OS (*p* = 0.0123). This effect could not be observed in group A.

A loss of 13q in group B was significantly associated with a longer OS (*p* = 0.0364). Again, this effect could not be observed in group A.

No further significant correlations regarding clinical, chromosomal and epigenetic data could be observed.

## Discussion

The focus of this study was to find new molecular markers for treatment response in GBM. Only a few previous retrospective and prospective studies have analyzed the combination of carmustine wafer implantation with the combined standard chemoradiation protocol for the treatment of newly diagnosed glioblastoma [26–35].

We report here the impact of carmustin wafer implantation together with the combined standard chemoradiation protocol in newly diagnosed supratentorial glioblastoma in adults. To overcome the limitations inherent to retrospective observational studies, we performed a confirmatory case matched analysis (*N* = 72). The data from our study confirmed previous trials suggesting that MGMT is a predictive marker for TMZ therapy response [11, 14–19]. We also found a significant correlation between MGMT methylation status and OS in our total collective. This significance vanished when the patients were stratified for treatment group A or B. This may result from the limited number per treatment cohort. Losses of chromosome 10 are among the most frequent in GBM [36–38]. Patients with additional carmustine wafer treatment and a loss of chromosome 10 showed a significantly longer OS than patients without that chromosomal loss. An explanation for this could be the MGMT gene locus on 10q21. A loss of this region results in a loss of MGMT expression and therefore ameliorate the treatment response of both TMZ and the local carmustin therapy. Wemmert et al. could show a similar effect regarding TMZ therapy alone [38]. In our trial this effect occurred only in patients receiving both TMZ/RT → TMZ regime and carmustin wafer implantation, but not in patients who received TMZ/RT → TMZ regime alone. Possibly other so far unknown gene loci might also play an important role.

Mutations and deletions of p15 and p16 are frequent genetic alterations in glial tumors [39–43]. p15 and p16 inhibits CDK4 and CDK6, therefore p15 and p16 act as tumor suppressors and lead to cell cycle arrest in the late G1 phase [33]. Previous studies indicated that a loss of expression, resulting from deletion, mutations or methylation of p15 and p16 is associated with a significantly worse prognosis for survival in glioblastoma [21, 38, 44, 45]. Our data supports this point of view, at least regarding p15. Interestingly patients administered to group B with methylated p15 showed significantly the shortest OS of all subgroups within our trial. The low number of only six p16 methylated tumors shows that p16 is not of significant impact on our collective. Considering the findings of the literature this is not surprising [21, 38, 44–46].

Another important finding of our study is the amplification of the region 4q12 as a prognostic marker in patients additionally treated with carmustine wafer. Patients in group B whose tumor showed this amplification had a significantly shortened OS. PDGFRα, a tyrosine kinase, is located in the region of 4q12. PDGFRα is known to play a major role in tumor angiogenesis by stimulation of cell growth [47, 48]. The exact mechanism of PDGFRα in vasculogenesis and tumor angiogenesis is yet unknown, but an overexpression of PDGFRα caused by gene amplification may result in more aggressive tumor growth.

This effect was not observable in group A. Maybe PDGFRα is not the only determinant gene influencing OS and due to the described tumor heterogeneity in GBM it did not reach a statistical significant level [24, 49, 50].

We also detected a better prognosis in group B if chromosome 13 or parts of chromosome 13 were lost. This is concordant with previous findings where a survival benefit in patients treated with alkylating agents was found, if chromosome 13 was lost [38]. A further genetic hotspot is the RB1 gene, which is located on 13q14.2. Maybe a loss of this gene influences the oncological behavior of tumor cells in such a manner that additional carmustine wafer therapy shows an improved impact on the clinical course. This effect could not be shown in the standard therapy group treated with Stupp regime. Hence a loss of chromosome 13 is maybe a prognostic marker for an ameliorated clinical course which would recommend the implantation of carmustine wafers.

Besides to the molecular findings described above, we found no significant survival benefit between group A and B in general. We think it is not recommendable to use additional carmustine wafer implantation in every single case. This is conclusive with the data of other clinical studies and the widespread use of carmustine wafers is highly controversial due to its debatable clinical impact. Pallud et al. could e.g., not show a long-term benefit regarding overall survival (OS) in a cohort of 354 patients [9]. This controversy is even more understandable if the clinical side effects of carmustine wafer implantation is taken into account. Especially operative wound infection and cerebral edema can be increased [10].

Therapy with carmustine wafers should be individually assessed for each patient. This also represents the current opinion in treatment guidelines, in general.

Overall, our findings suggest that carmustin wafer implantation in combination with maximal safe resection, followed by combined standard chemoradiation protocols, is a promising treatment option for patients with supratentorial glioblastoma harboring MGMT promoter methylation.

## Conclusion

A clinical benefit for the widespread use of additional carmustine wafer implantation could not be found. However, carmustine wafer implantation shows a significantly improved overall survival if chromosome 10 and particularly 10q or chromosome13 are deleted. In cases of 4q12 amplification and in cases of a methylated p15 promotor, the use of carmustine wafers is especially not recommended.

The MGMT promoter methylation is a strong prognostic Biomarker for benefit from temozolomide and BCNU chemotherapy.

Therefore we propose to use BCNU wafers in a second line therapy, when the chromosomal and epigenetic data from the primary tumor are available. However, owing to the small number of patients these findings would need to be corroborated in lager patients cohorts.

## Abbreviations

CGH: Comparative genomic hybridization
TMZ: Temozolomide
WHO: World Health Organization
